# Antioxidants in Fish Sperm and the Potential Role of Melatonin

**DOI:** 10.3390/antiox10010036

**Published:** 2020-12-31

**Authors:** Francisca Félix, Catarina C. V. Oliveira, Elsa Cabrita

**Affiliations:** Centre of Marine Sciences (CCMAR), University of Algarve, Campus Gambelas, 8005-139 Faro, Portugal; ffmelo@ualg.pt

**Keywords:** antioxidants role, oxidative stress, melatonin, spermatozoa

## Abstract

In recent years, the effects of novel antioxidants have played an important role in the research focusing on fish cell protection. As food demand grows, aquaculture production becomes more intensive, and fish are more exposed to oxidative stress conditions, like high densities, temperature shifting, frequent fish handling and samplings, and prophylactic or disease treatments, which expose fish to a different environment. Particularly in reproduction, germ cells lose antioxidant capacity with spermatogenesis, as spermatozoa are more prone to oxidative stress. Antioxidants have been used in a variety of fish physiological problems including in reproduction and in the establishment of cryopreservation protocols. From the most used antioxidants to natural plant food and herbs, and endogenously produced antioxidants, like melatonin, a review of the literature available in terms of their effects on the protection of fish spermatozoa is presented here in a classified structure. Several direct and indirect approaches to improve gamete quality using antioxidants administration are mentioned (through feed supplementation or by adding in cryopreservation media), as well as factors affecting the efficiency of these molecules and their mechanisms of action. Special attention is given to the unclear melatonin pathway and its potential scavenger activity to prevent and counteract oxidative stress damage on fish spermatozoa.

## 1. Introduction

Oxidative stress represents one of the principal damage suffered by fish spermatozoa. Many are the external factors behind this damage, but nutrition in aquaculture plays an important role in the control of oxidative events by providing adequate compounds that can be transported into the germline. Fish sperm is particularly prone to suffer oxidative damage due to the high content of polyunsaturated fatty acids (PUFA) present in their membranes. Oxidative stress has been shown to reduce several spermatozoa functions, impairing sperm motility, cell viability and functionality, and, at a later time, DNA integrity [1]. The causative links between oxidative stress and sperm function have subsequently been confirmed in spermatozoa of several mammalian species (reviewed recently by [2]). Extensive literature has reported the effect of reactive oxygen species (ROS) in germ cells in different species, including fish, and how this oxidative stress can be counteracted by a balance between antioxidative and pro-oxidative compounds (see reviews [3,4]). As a biological response to oxidative stress, seminal plasma has evolved to one of the most powerful antioxidant fluids known, being its antioxidant capacity estimated to be higher than blood [2]. However, several factors may contribute to an imbalance of these compounds, and especially in intensive farming, where fish are exposed to oxidative stress-promoting conditions, like high densities, temperature shifting, frequent handling and samplings, and prophylactic or disease treatments.

As mentioned, antioxidants provided in the diet have a pivotal role in this balance and are special important during the periods of gametogenesis and spermiation. In fish, the importance of antioxidants protecting sperm from oxidative damage has been highlighted by several authors [5,6,7,8,9], but a comprehensive revision of their effects was not presented exhaustively focusing on all types of antioxidants reported in the literature and demonstrating their differential effect and species specificity. In reproductive tissues, antioxidants have been used as supplements in the extender media to protect germ cells during cryopreservation. This review gives an overview of the types of antioxidants used in fish sperm and its impacts on sperm quality biomarkers, namely in lipid peroxidation, protein oxidation, DNA impairment, cell viability, fertilization capacity, and in all motility sperm traits that can affect the success of the reproductive methodologies of modern hatcheries. Different approaches with importance for the aquaculture industry are explored, from dietary experiments with impact in the development of new broodfeed products for feed companies to the applicability of antioxidants on the development of new sperm extenders or fertilizing solutions used in reproductive management techniques. The different types of antioxidants are addressed in a categorized way and a special attention is given to melatonin (MEL) due to the few studies conducted in fish sperm. This molecule has a huge importance and a variety of functions on the biology of all organisms, from bacteria to plants and animals. Moreover, it is endogenously produced by fish, so its antioxidant proprieties are extremely natural, which constitutes a perfect opportunity to be explored regarding its application on fish spermatozoa protection. The scope for research about MEL is on a dedicated section. The potential of MEL, not only but mainly as an antioxidant, can have a direct impact on aquaculture production, especially within broodstock facilities that uses photic manipulation to induce broodstock spawning, smoltification, or early maturation [10]. As an endogenously produced hormone, it can trigger some of the above biological functions. On the other hand, as an antioxidant, it can be used either to enhance the overall welfare of fish in captivity or used as an exogenous supplement to improve specific reproductive traits, such as fish sperm quality [11]. The total knowledge of endogenous MEL production would be of a huge interest for aquaculture, but first, the scientific community needs to comprehend its mechanisms of action, production sites, and its influence on fish biology, in particular at a gonadal level. Here, we summarize some of the most recent findings regarding fish sperm.

## 2. Spermatozoa Susceptibility to Reactive Oxygen Species and the Counteract Effect of Antioxidants

**Figure 1:**
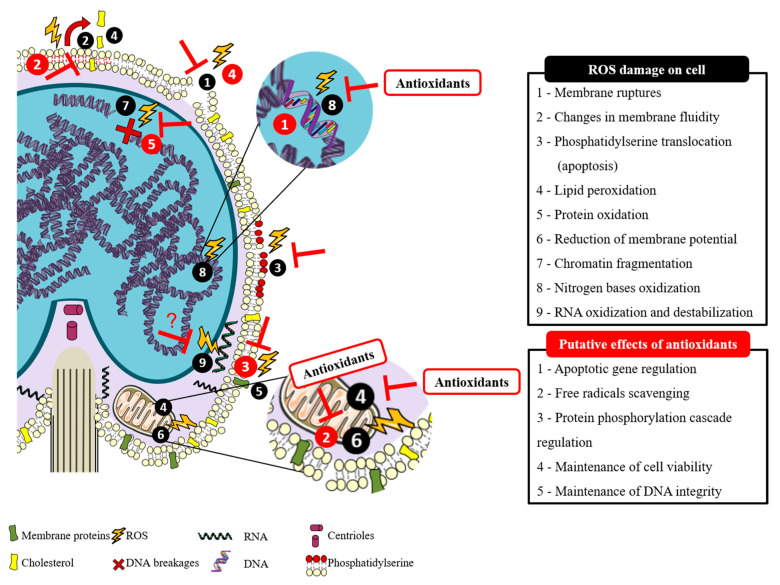
Putative effects of antioxidants on counteracting reactive oxygen species (ROS) damage in fish sperm. Adapted from Cabrita, et al. [1].

The paternal genome transference into the oocyte is the specific purpose of spermatozoa. During spermatogenesis, the content of cytoplasm is drastically reduced, and the cells become differentiated spermatozoa, occupied mainly by DNA on the commonly named sperm head [3]. Through this process, the spermatozoa intracellular antioxidant capacity decreases, and the defense system against oxidative stress depends mainly on the seminal plasma [5]. For species with external fertilization, like fish, these unicells need to survive and exert their function on a very different environment after losing their main protection medium: the seminal plasma, which contains antioxidant components that confer protection against Reactive Species (RS) [12]. There are two main types of Reactive Species: the above-mentioned ROS and Reactive Nitrogen Species (RNS). Both are metabolic by-products that can be generated during normal cellular oxygen metabolism, but can also be involved in oxidative stress situations [13], like fish handling, temperature shifting, or environmental pollution. Moreover, in artificial reproductive techniques, like cryopreservation, oxidative stress has been considered the main reason for spermatozoa malfunctions [14]. Although ROS production is not a specific threat of the reproductive cells [15], according to Cabrita, et al. [1], fish spermatozoa membrane is a perfect target for ROS, which may also cause lipid and protein peroxidation, damage on midpiece and axoneme, DNA fragmentation and mitochondrial impairment (Figure 1) [16,17]. All this damage can consequently lead to a decrease in sperm motility and fertilization capacity [5,14]. However, recent literature states that ROS have a bivalent role in sperm function [3]. In other words, levels of ROS that do not surpass the normal biological records are necessary for the correct function of sperm [18], being mild levels important to intracellular reactions associated with the fertilization capacity, and high levels responsible for embryo miscarriage [3]. To prevent the cellular damage provoked by ROS, the seminal plasma has a specific defense system composed of enzymatic and non-enzymatic antioxidants able to scavenge the excess of ROS in order to achieve some biological balance between the beneficial effects of ROS and the damaging oxidative stress [19]. Recently, several studies have been developed to improve fish sperm oxidative stress defense system by using different antioxidant supplementation strategies.

Antioxidants are substances capable of preventing or diminishing the oxidation of other molecules [20] (Figure 1), by neutralizing free radicals before they interact with biological targets [21]. In the last decades, researchers have been investigating the potential effects of antioxidants on human and animal health. It has been reported that immunocompetence, fertility, growth, ageing, and sexual signals are positively affected by the action of antioxidants [22], as well as some sperm properties [23]. Fish seminal plasma and spermatozoa composition include a different range of antioxidants: some are enzymatic like superoxide dismutase (SOD), catalase (CAT), glutathione peroxidase (GPx), glutathione reductase, others are vitamins as α-tocopherol (vitamin E), ascorbic acid (vitamin C) and there are also low molecular weight antioxidants, like glutathione (GSH) [14,24]. Several authors have reported that sperm quality parameters, such as motility and viability, can be positively affected by broodstock nutrition [16,25,26], which can be supplemented with a great variety of antioxidants, like those that naturally occur on sperm or others.

## 3. Fish Sperm Antioxidant System and Supplementation: Classification and Identification

Different antioxidant classifications have been established around the world. Catoni, et al. [20] reviewed literature on fish, birds, and mammals, and divided antioxidants into endogenous and dietary antioxidants, and the last ones into focal antioxidants, which are intrinsically important for a specific trait, and non-focal antioxidants that indirectly influence traits. Mironczuk-Chodakowska, et al. [13] go a bit further and not only divided them into endogenous and exogenous, but also considered endogenous ones as the products of normal metabolism, which can be enzymatic or non-enzymatic. Moreover, they also added that exogenous antioxidants (dietary antioxidants) such as vitamins, minerals, and polyphenols, can influence and act synergistically with the first ones.

**Figure 2:**
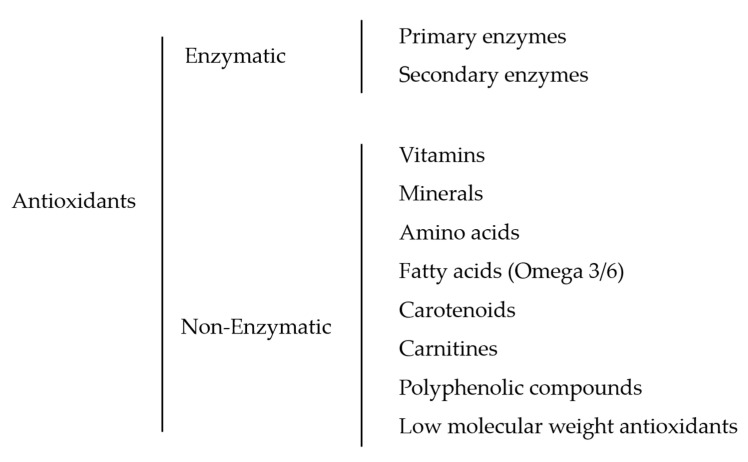
Classification of the reviewed fish sperm antioxidants.

It is generally known that antioxidants can be classified according to their biochemistry, occurrence, mechanism of action, solubility, and kinetics [21], but the most simple and widely accepted classification is the division of two major groups of antioxidants according to their mechanism of action: enzymatic and non-enzymatic [22]. In this review, we have tried to classify and identify all the antioxidant substances used to enhance the quality of fish sperm traits. As a result, the hierarchy of the antioxidants used is illustrated in Figure 2.

### 3.1. Enzymatic Antioxidants

According to their mechanism, enzymatic antioxidants can be primary or secondary, but literature also considers multiple-function antioxidants, which have both primary and secondary functions [23]. Hermund [24] described primary enzymes as responsible for fighting lipid oxidation once they can stabilize free radicals by direct reactions. There are three main primary oxidant scavengers: SOD, which catalyzes superoxides into hydrogen peroxide and oxygen, GPx, which has the same function, and CAT, which participates in the previous pathway and degrades hydrogen peroxide into water and oxygen [19]. On the same review, Hermund [24] defined secondary enzymes as preventive antioxidants, since they act indirectly on lipid oxidation and can also have a synergistic effect by regenerating primary antioxidants, like glutathione reductase, which is responsible for regenerating GSH and its action is triggered by oxidative stress and low levels of GSH in the organism [14]. Glucose-6-phosphate dehydrogenase and glutathione-s-transferase are also examples of secondary enzymes [22], although the number of studies that focus on secondary enzyme supplementation and their effects on fish sperm quality are not significant so far. On the contrary, all of those above-mentioned primary enzymes have been used to in vitro experiments and as supplements for fish sperm extender mediums, either for short- and long-term storage [25,26].

Lahnsteiner and Mansour [27] started comparing the sperm antioxidant defense system of different teleost fish: Salmonidae, Percidae, Lotidae, and Cyprinidae, and the concentration of different enzymatic antioxidants revealed only minor interspecific differences in terms of qualitative and quantitative patterns. Among other results, concentrations of glutathione reductase, a secondary enzyme, were low in bleak (*Alburnus alburnus*), burbot (*Lota lota*), perch (*Perca fluviatilis*), and brown trout (*Salmo trutta*) semen and SOD activity was higher when concentrations of other enzymes and metabolites were low or variable. Only CAT was not detectable in seminal plasma and spermatozoa of bleak despite the positive effect on sperm motility and membrane integrity in all other species [27]. Similarly, Hagedorn, et al. [28] had good results using CAT to mitigate the nocive effects of the induced ROS (through xanthine-xanthine oxidase) on zebrafish (Danio rerio) sperm, with motility and viability being maintained or improved over the control levels. However, in a cryopreservation trial, despite the positive effect of CAT addition to the extender medium, causing an improvement of sperm motility after thaw, this enzyme was not able to reduce ROS production after cryopreservation. Contrarily, CAT and GPx had no positive effect on rainbow trout (*Oncorhynchus mykiss*) sperm after thaw on an experiment performed by Kutluyer and his team. Moreover, Kutluyer, et al. [29] reported that thawed sperm motility rate and its duration were positively affected by SOD supplementation in the extender, but no effects were verified regarding fertilization capacity and hatching rate. Regarding more ancestral fish species, Li, et al. [25] tested the efficiency of a variety of antioxidant supplementation on sperm extender medium and, concerning oxidative defense enzymes, CAT demonstrated to be very efficient, maintaining spermatozoa membrane and acrosome integrity in three sturgeon species: Siberian sturgeon (*Acipenser baerii*), Chinese sturgeon (*Acipenser sinensis*), and Dabry’s sturgeon (*Acipenser dabryanus*), even at low concentrations.

### 3.2. Non-Enzymatic Antioxidants

There is a wide range of non-enzymatic antioxidants and some characteristics of their biochemistry, which are important to understand their availability on tissues, potency, and mechanism of action [20]. It is possible to find lipophilic and hydrophilic antioxidants in the cells. However, lipophilic antioxidants such as vitamin E and most carotenoids are found in cytoplasm, whereas hydrophilic antioxidants such as vitamin C and polyphenolic substances are found in cell membranes [30]. Having those characteristics in mind, a great variety of non-enzymatic antioxidants have been used by different research teams as a supplement to increase the oxidative defense system of fish sperm.

#### 3.2.1. Vitamins

Among other vitamins, vitamin C and E are the most critical non-enzymatic antioxidants present in seminal plasma due to their natural abundance in the medium and their important role in fish reproduction [31]. Vitamin C, also known as ascorbic acid, is a water-soluble antioxidant responsible for scavenge the oxidative effect of radicals from different sources [19], like hydroxyl, superoxide and hydrogen peroxide. Most importantly, ascorbic acid oxidized form does not cause damage to the cells [14]. Moreover, it acts as a cofactor for many enzymes, the most representative ones being involved in the synthesis of collagen, proteoglycans, and other components of the intercellular matrix, and participates in the recycling of oxidized vitamin E [30,32]. On the other hand, fat-soluble vitamin E, also known as α-tocopherol, is responsible for protecting cell membranes, avoiding lipid peroxidation and DNA damage (Figure 1), thus improving the function of other antioxidants [32]. According to Halliwell and Gutteridge [30], some cold-water species, like salmon, can acquire a marine-derived tocopherol (MDT) from the plankton they eat [33] that might permit more mobility within the membrane, which increases the antioxidant capacity at low temperatures.

The interaction between vitamin C and E have been reported in several in vivo studies, including in fish species [34]. Among all studies, the most positive results were observed in a study performed by Figueroa, et al. [14] in Atlantic salmon (*Salmo salar*) sperm, in which the addition of ascorbic acid and α-tocopherol to the freezing medium decreased the superoxide production and lipid peroxidation and increased the spermatozoa membrane potential and motility, which culminated with a higher fertilization capacity. This cascade of positive effects could be due to the synergic effect of the simultaneous use of both vitamins. In other types of studies, in which they have just tested the effect of isolated vitamins, there were also good results for some specific parameters. Martínez-Páramo, et al. [9] demonstrated that vitamin C or E can have a positive effect on European seabass (*Dicentrarchus labrax*) sperm once motility parameters, such as total motility, velocity, and linearity, increased in thawed sperm that was cryopreserved with mediums supplemented with 0.1 mM α-tocopherol and with 1 mM ascorbic acid, but lipid peroxidation, protein oxidation, and viability did not show signs of improvement. These results are in agreement with those achieved in rainbow trout by Kutluyer, et al. [29], in which supplementation of freezing medium with α-tocopherol (2.0 mmol/L) increased the thawed sperm motility and duration, but fertilization rate was not affected by the antioxidant supplementation, while ascorbic acid (0.5 mmol/L) did not show any positive effects.

So far, reviewed results are already pointing to some species-specific effect, depending on the type and dose of antioxidants used [5,28]. Vitamin E supplementation on extenders mediums revealed different positive effects on thawed sperm from different species. In Senegalese sole (*Solea Senegalensis*), α-tocopherol increased the percentage of progressive spermatozoa and velocity [16], while in Artic char (*Salvelinus alpinus*) it increased the seminal plasma antioxidant potential and inhibited lipid peroxidation [6]. It is also possible to find vitamin C experiments with this inequality in results. In Siberian, Chinese, and Darby’s sturgeon, the addition of 0.5 mg/mL of vitamin C to the cryoprotectant medium showed protective effects on acrosome and membrane integrity, but no effect on fertilization rate [25], whereas in Russian sturgeon (*Acipenser gueldenstaedti*), extender supplementation with 0.1 M of vitamin C not only increased motility and fertilization ability, but also decreased DNA mutations in the embryos obtained after fertilization with cryopreserved sperm [35]. In a study performed by Liu, et al. [26] with red seabream (*Pagrus major*) sperm, dose-dependence was clear on the obtained motility results, in which despite the positive effects of vitamins A, C and E supplementation in the cryoprotectant medium, higher levels of vitamin A and E (50 mM, 100 mM) did not improve sperm motility. There are also some studies indicating that antioxidant supplementation had no effect on sperm quality, like in gilthead seabream (*Sparus aurata*) and European seabass, where Cabrita, et al. [5] states that neither vitamin C, E nor other amino acids used had had effects on motility or viability.

The majority of studies focus on the action of vitamins supplemented to the extender mediums, but long-term studies would be necessary to determine whether antioxidants supplementation through diets could have different results on enhancing fish sperm quality. Lee and Dabrowski [34] studied the long-term effects and interactions of vitamin C and E on growth and reproduction of yellow perch (*Perca flavescens*), and found that 32 weeks supplementation improved growth rate. In what semen quality is concerned, it would take just one maturation cycle to observe the positive effects of vitamin C (higher viability, male gonadossomatic index, and hatching rate) in fish fed with vitamin C. Interestingly, sperm levels of α-tocopherol were higher in fish fed with ascorbic acid rather than α-tocopherol. Thus, the authors concluded that vitamin C may spare sperm vitamin E depending on vitamin E reserves on tissues. Martínez-Páramo, et al. [7] tested both types of supplementation together (nutrition and extender supplementation) and concluded that supplement diets with vitamins could enhance the antioxidant system of European seabass and gilthead seabream and afterwards, together with extender supplementation, improve sperm quality after cryopreservation on both species, despite the species-specific effects observed.

#### 3.2.2. Minerals

Macro and microminerals are essential nutrients involved in structural and functional processes, respectively. In the organism, phosphorus (P), calcium (Ca), and magnesium (Mg) are examples of macrominerals responsible for the formation of hard structures (scales and bones), while zinc (Zn), manganese (Mn), and selenium (Se) are microminerals important for metalloenzymatic processes [36].

Selenium (Se) is indispensable for many biological molecules, specifically for selenoproteins and selenoenzymes, like GPx, whose expression and activity improve with the inclusion of Se in the diet [37,38]. Although the optimal levels of Se are still unclear, its addition in the feeds can strongly benefit fish antioxidant and immune system [39]. Once in culture, Se intake depends on the feeds, while wild fish can take it from the surrounding medium [40]. In aquatic systems, fish are also exposed to different compounds that may be prejudicial because of the direct absorption and accumulation on tissues [41]. In African catfish (*Clarias gariepinus*) exposed to mercury (Hg), Se did not avoid the Hg accumulation on testicular tissues, but sperm quality recovered in fish also exposed to Se (water dissolved), as well as total antioxidant capacity and GPx activity [42]. Se is available in inorganic, organic, and nano form, but most research in fish focus on the addition of inorganic Se in fish diets [40]. Wischhusen et al. (2019) tested different types of Se supplementation (sodium selenite or hydroxy-selenomethionine) on rainbow trout broodstock and proved a maternal transference of Se to the progeny due to higher levels in the oocytes in both Se-supplemented groups. Organic Se led to higher levels of progeny vitamins E and C. In sperm, however, Se concentrations were not different between supplemented diets or control. It is also important to mention the synergic effect of Se combined with other trace elements or nutrients, which can induce or have no effect on Se toxicity, namely in the case of simultaneous use of Se and Zn, or Se and vitamins [40]. In a study performed with Senegalese sole, Beirão, et al. [16] observed the GPx Se-dependent effect by the observation of higher GPx activity in blood plasma on fish fed with Se+vitE diet whom, at the reproductive tract, showed an improvement on sperm motility parameters (progressive motility and curvilinear velocity) due to Se+vitE intake. Martínez-Páramo, et al. [7] studied the effect of diet supplementation with Se, Zn, or vitamins (C and E) on European seabass and gilthead seabream sperm quality and found higher Se concentration in sperm of seabass fed with Se diet. Regarding Zn diet, it improved neither Total Antioxidant Status (TAS) nor Zn levels in sperm from both species. On the cryopreservation experiment, the same authors, Martínez-Páramo, et al. [7] also detected species-specific effect of minerals, as it was observed in vitamins, once Se and Zn reduced seabass sperm lipid peroxidation, but only vitamins had the same effect on seabream sperm. Se is essential, but becomes toxic at concentrations near to the fish nutritional requirements [43]. An investigation with Nile tilapia (*Oreochromis niloticus*), performed by Chen, et al. [39] revealed that Se supplementation is beneficial at low levels to the fish organism, but toxic when added at high concentrations (>12 µg/g) to the diets.

Zn is another essential mineral with a great variety of functions, including in reproduction, which can be explained by its cofactor role in more than 200 enzymes spread all over the organism [44]. It is the second most abundant mineral in the body, but at the same time, it cannot be stored, as its concentration is dependent on dietary intake [45]. It is involved in the correct spermatozoa DNA condensation and decondensation [37] and inhibits the membrane-bound with oxidative enzymes, such as NAD(P) oxidase, acting as a membrane stabilizer [46] and as a potent antioxidant preventing lipid peroxidation [3], which by itself provoke changes in membrane morphology, increasing its fluidity and decreasing its functionality [47] (illustrated in Figure 1.) Zn also participates in hormone synthesis and secretion (Luteinizing Hormone and Follicle-Stimulating Hormone), gonadal differentiation, testicular growth, spermatozoa maturation, and fertilization [44]. As with other minerals, Zn has many sources and, depending on them, it can have different functions [48]. Still, all of them can be added to the diet to meet the nutritional requirements of fish [49], whether organic, mineral or Zn nanoparticles. A study performed with rainbow trout male broodstock, tested the effect of three sources of Zn (mineral, nanoparticles, or organic) supplemented in the diets [50]. With this experiment, the authors found that mineral Zn had a positive impact on sperm quality and reproductive performance, due to the obtained high rates of fertilization, eyeing, hatching and offspring survival. In addition, sperm motility duration and spermatocrit percentage were higher in all Zn treatments compared to control [50]. This influence on viability and motility is due to the Zn action on lipid catabolism, functioning as the source of energy for spermatozoa motility [45]. A study performed on catfish (*Pangasianodon hypophthalmus*) [51] revealed that feed supplementation with 200 mg/kg of Zn could improve sperm concentration and volume over 50% and percentages of sperm motility and viability were also significantly higher than with other treatments. In a different experiment, Aripin, et al. [52] supplemented the diets with a combination of melatonin and zinc and showed that this mixture could reduce the abnormalities on spermatozoa and increase some kinetics parameters, such as sperm motility, concentration, and lateral amplitude. Despite the good results obtained in some studies, the few experiments performed with fish sperm and the absence of an appropriate control in some trials does not allow for further discussion on the effect of minerals intake on fish sperm quality and reproduction.

#### 3.2.3. Amino Acids

Other components that can be used to enrich broodstock nutrition are amino acids, whether essential or non-essential, used individually or combined, which can directly or indirectly have an impact on fish health [53]. The biological functions of amino acids are wide: they are regulators of gene expression and protein phosphorylation cascade (Figure 1) [54], precursors for a variety of molecules, such as steroids, glycogen, nucleotides, lipids and low molecular weight nitrogenous substances [54,55] and participate in many other pathways, including fish antioxidant defense system [53]. Specifically on teleost fish, amino acids are one of the seminal plasma key components which impact some spermatozoa traits, differing qualitatively and quantitatively between species, like in gilthead seabream, perch, rainbow trout, and common carp (*Cyprinus carpio*) [56,57]. Butts, et al. [58] studied the effect of enhanced feeds with modified amino acid composition on European eel (*Anguilla anguilla*) sperm biochemistry and reproductive performance and found that certain amino acids can regulate milt biochemistry. Furthermore, amino acids intake has a direct influence on fish sperm and can promote some kinetic traits. Once they are naturally found at high concentrations in sperm, and the antioxidant capacity of some is undeniable, amino acids have also been used to supplement cryopreservation extenders in order to better protect the cells against cryodamage [59]. Since sperm dilution in the extenders exposes even more of spermatozoa to oxidative processes due to a decrease in the natural antioxidant defense system [5], these additions could be crucial. Interestingly, the species-specific effect also prevails when performing amino acids supplementation, as it was demonstrated with cysteine in two different studies using common carp and South American silver catfish (*Rhamdia quelen*), two of the most common freshwater species exposed to the same dose of cysteine (2.5, 5, 10 and 20 mM), but with completely different results. In catfish, the addition of 5, 10 and 20 mM of cysteine to the extender medium provoked protein and lipid oxidation damage to the post-thaw sperm [60], while common carp sperm motility, DNA damage, and fertilization were positively affected with the increase in cysteine concentration, being 20 mM the treatment with better results [59]. An increase in carp sperm motility was also observed when 1 mM of cysteine was added for short-term storage (up to 24 h) at 4 °C [61]. Cysteine is a sulfur-containing amino acid that enhances GSH production both in vitro and in vivo, which has a direct impact on ROS [62,63], being involved in a general antioxidant system of the organism [64]. Stejskal, et al. [65] measured the cysteine content in some fish sperm samples, using high performance liquid chromatography with electrochemical detector (HPLC-ED), and the lowest content of cysteine was found in Siberian sturgeon (55 ± 8 µg/mL) and sterlet (*Acipenser ruthenus*) (62 ± 9 µg/mL), and the highest in the samples of pikeperch (*Sander lucioperca*) (251 ± 38 µg/mL) and perch (281 ± 42 µg/mL). These results are in accordance with the results obtained by Li, et al. [25] on a cryopreservation assay, wherein he concluded that the addition of cysteine (N-acetylcysteine, NAC) to the extender medium had no effect on post-thaw quality semen parameters in any of the three sturgeon species analyzed (Siberian, Chinese and Darby’s). In addition, the authors stated that a concentration higher than approximately 9.8 mM in the cryopreservation medium was associated with lower spermatozoa motility, acrosome and membrane integrity and even lower fertilization rate.

Like cysteine, methionine is another essential amino acid that is a precursor of GSH and is used in different experiments to reduce sperm ROS production [66]. It participates in protein and polyamine synthesis (spermine and spermidine) that take part in cell division events, and is the most important methyl group donor for DNA methylation reactions [67]. Methionine is present both in seminal plasma and spermatozoa of rainbow trout, but only in seminal plasma of common carp, although this amino acid plays an important role in the antioxidant defense system of both species, having a positive impact on sperm viability [56]. Regarding cryopreservation trials testing methionine supplementation in the extender, there was some disparity in the results obtained. Lahnsteiner, et al. [68] tested the addition of different types of antioxidants to the extender, and despite reduced methionine increased the velocity of thawed sperm from brook trout (Salvelinus fontinalis), the conclusion was that antioxidants supplementation is not recommended for routine sperm cryopreservation procedures with brook and rainbow trout. Three years later, Kutluyer, et al. [29] performed a cryopreservation assay using the same antioxidant supplements and, among others, methionine increased the motility rate and the duration of rainbow trout thawed sperm. Yet, fertilization and hatching rates were not affected by any antioxidant. In brown trout, Lahnsteiner, et al. [17] performed an in vitro incubation study and both reduced and oxidized methionine were present in both seminal plasma and spermatozoa, and had positive effects on spermatozoa membrane integrity and motility rate, though membrane lipid peroxidation did not decrease, remaining unclear the metabolic pathway of methionine and the importance of this amino acid to the antioxidant system of brown trout. In accordance with these results, another study from the same group with sperm from burbot, brown trout, perch and bleak, revealed that oxidized methionine had a positive impact on sperm viability from all species and reduced methionine only impacted motility rate and spermatozoa membrane integrity of brown trout, perch [27]. Nevertheless, looking to different species, it is possible to find more consistent results using different amino acids. Kutluyer, et al. [66] performed a cryopreservation experiment using methionine or taurine on the extender of goldfish (*Carassius auratus*) sperm, and the best results in terms of post-thaw motility parameters and DNA damage were obtained using 1.5 mM methionine. A second trial using a different amino acid, taurine, obtained similar positive effects with 4 mM added to the extender [69].

Taurine and hypotaurine are sulfuronic amino acids that have many physiological functions, including fat digestion, membrane stabilization, antioxidation, osmoregulation, modulation of ion flux, control of calcium homeostasis and are involved in the visual, neural, and muscular systems [70,71]. Moreover, some studies indicate that taurine is present in the reproductive tract [72] and can regulate the rate of ROS production by mitochondria, acting indirectly on oxidative stress, although its mechanism of action is still unclear [71,73]. As with other amino acids, there are effective and non-effective results on post-thaw fish sperm quality when cryopreservation medium was supplemented with taurine. In rainbow trout, none of the three concentrations tested (50, 75, and 100 mM) increased post-thaw sperm quality [72]. Studies performed by our group showed similar results when taurine or hypotaurine were added to the freezing media, with no improvement on motility or viability parameters in European seabass and gilthead seabream. However, in seabream, both amino acids decreased DNA fragmentation and 1 mM of taurine improved spermatozoa motility [5]. On the same line of research, Martínez-Páramo, et al. [74] obtained similar results with European seabass, wherein the viability of the cell was not affected either by taurine nor hypotaurine, together with lipid and protein oxidation. Contrary to previous results on seabass, 1 mM of hypotaurine improved total sperm motility and 1 mM of taurine resulted in higher sperm velocity and linearity, and both amino acids reduced DNA fragmentation on spermatozoa. As reviewed so far, taurine can have impacts on some sperm quality parameters, but there are no representative indications of preventing oxidative damage in fish sperm. Only in Japanese eel (*Anguilla japonica*) taurine was found to promote superoxide activity (SOD) in the testis [75], but further investigation is necessary to understand if this amino acid can impact ROS production. In accordance, the results from Liu, et al. [26] with red seabream revealed that thawed sperm motility improved with 50 mM taurine in the extender, but no alterations were verified regarding antioxidant enzymatic activity and lipid peroxidation (MDA). A more recent study with amberjack broodstock diets enriched with taurine, histidine and proteins has revealed that histidine has a great importance in embryo and larval development and taurine in fecundity and fertilization rates, although optimal dietary levels of those amino acids were not identified [76]. Regarding histidine, its dietary requirements and metabolism are influenced by environmental and endocrine factors [70], but there is no knowledge of its influence on fish reproduction and sperm antioxidant status until now.

Tryptophan is another essential amino acid, which has already been tested as an extender supplement in different species of trout, either for long- or short-term storage of sperm. This is an aromatic amino acid, involved in many metabolic functions and crucial for protein synthesis [77]. Kutluyer [78] established the best concentrations of tryptophan for sperm cryopreservation of three trout species: rainbow trout, coruh trout (*Salmo coruhensis*), and Anatolian trout (*Salmo rizeensis*), wherein the best results were obtained with 0.5, 2 and 5 mM, respectively. In this study, tryptophan had impact on spermatozoa motility, fertilization and hatching rates. One year later, Kocabas, et al. [79] used the same amino acid for a short-term storage experiment with sperm from rainbow and brook trout and the results were in accordance with the previous, being 0.5 mM the concentration of tryptophan that better impacted sperm motility parameters (6 and 11 days of motility for rainbow and brook trout, respectively). One of the most important pathways of tryptophan is its conversion to serotonin, a neurotransmitter, and melatonin, an antioxidant [70]. In another type of study, Salamanca, et al. [80] analyzed the long-term effect of tryptophan on stress and metabolism of Senegalese sole and concluded that light stress can be attenuated by tryptophan supplementation on feed. Despite the amino acids already mentioned and tested on fish sperm quality, arginine, glutamine, leucine, and proline are examples of functional amino acids that play important roles in key metabolic pathways, such as growth, immunity and reproduction [54], which could be explored as antioxidative protectors. Proline was demonstrated to be present in rainbow trout tissues by Dabrowski, et al. [81], and nowadays it is considered as a conditionally essential amino acid for fish in early live stages and perhaps in adult forms as well [70], but so far, it was not investigated whether it has a role in fish reproduction and gamete quality. Moreover, Arginine is known for its crucial role in regulating endocrine and reproductive functions, among many others, and fish have high requirements of dietary arginine [70,82], but its role as a scavenger of ROS or as a protective agent of spermatozoa was not yet investigated in fish.

#### 3.2.4. Omega-3 Fatty Acids

Omega-3 are polyunsaturated fatty acids (PUFAs), such as linolenic, eicosapentaenoic, and docosahexaenoic acids [83] that incorporate into spermatozoa plasma membrane, helping the functionality of the cell, not only by conferring fluidity, but also through their anti-inflammatory and antioxidant proprieties [37]. Few studies describe the effect of PUFA and omega-3 supplementation in the diet and its influence on sperm quality even though it is known that fatty acids are a major component of gametes as they play an important role in sexual maturation and reproductive performance of fish [31]. In addition, for species such as rainbow trout [84] and European seabass [85], the presence of some fatty acids on sperm depends on the dietary fatty acid intake, and an imbalanced feed composition can compromise the spawning quality [31]. The results of Koprucu, et al. [86] with rainbow trout sperm are in accordance and revealed that omega-3 can have a positive impact on fish sperm. In his study, the author demonstrated that fish fed on a diet supplemented with 2% anchovy oil had higher amounts of PUFA on sperm and had lower lipid peroxidation (MDA) levels. Moreover, even with the lower concentration (1% anchovy oil), sperm volume, density, and motility parameters were also enhanced up to 30 days post-feeding [86]. These effects were not only demonstrated in fish but also in mammalian species. A study performed on dog (*Canis lupus familiaris*) sperm showed that after 90 days of animals fed with salmon oil omega 3 and 6 (ratio 10:1) there was a prolonged increase in sperm concentration and motility during the following two months after ceasing the supplemented feeding [87]. Also, on boars (*Sus scrofa*) fed with omega-3 supplement, total sperm per ejaculate and duration of ejaculation were positively affected by treatment [83]. Regarding the effect of PUFA on fish sperm, the dietary regime with higher content of PUFA significantly affected common barbel (*Barbus barbus*) sperm morphology and velocity [88]. A positive synergic effect of fatty acids (DHA) and other antioxidants were also identified on Senegalese sole by our group, wherein diet with higher amount of PUFA + Vitamin C + Se showed to improve sole sperm quality parameters, such as progressive motility and velocity [16].

#### 3.2.5. Carotenoids

The major sources of carotenoids are plants and microorganisms [21]. These pigments, such as α-carotene and β-carotene, have a double-bounded structure and the chemical ability to react with free radicals, which confer them antioxidant proprieties [89]. Among all carotenoids, the most important for vitamin A generation is β-carotene [30]. Among other known carotenoids, such as xanthophyll, zeaxanthin, lutein, and lycopene, the last one has been recently studied due to its ability to fight some toxicity and important diseases, like cancer, heart disease, blindness, atherosclerosis, and multiple sclerosis, likely acting via prevention of lipid peroxidation [89,90]. Lycopene is a red pigment that naturally occurs in tomatoes and other red fruits [91] and is one of the most effective antioxidants among the carotenoids. It has the ability to stimulate the activity of SOD, GPx, and glutathione reductase, all important endogenous antioxidative enzymes [92]. In a study with Nile tilapia Abdel-Daim, et al. [93] provoked intoxication of blood and tissues with nano-zinc oxide (ZnONP) and proved that antioxidative proprieties of lycopene could ameliorate the nocive effects of ZnONP and restore the activity of SOD, CAT, and the levels of GSH in intoxicated fish. Similar results were obtained by other groups using dietary lycopene to attenuate the blood and tissues toxicity effects of endosulfan and diazinon in Nile tilapia [94,95], carbofuran in African catfish [96] chlorpyrifos, cypermethrin and deltamethrin in common carp [97,98,99] and oxytetracycline in rainbow trout [100]. Moreover, in some experiments, lycopene produced better effects combined with other natural antioxidants [93], which is in accordance with Amarowicz [91], who states that mixtures of carotenoids have higher antioxidative potential than any compound alone. Still, the mechanism of action of the antioxidant proprieties of carotenoids is not very clear [101]. The study with astaxanthin feed supplementation in rainbow trout by Jensen, et al. [102] was an example of that. Results revealed that astaxanthin is more effective to protect against the very early stages of lipid oxidation, while α-tocopherol, another lipophilic antioxidant focuses its antioxidative protection in more advanced stages of lipid oxidation. Despite the existing research of carotenoids and its antioxidative properties in fish, until now, only β-carotene was tested in fish sperm. The results obtained by Kutluyer, et al. [29] did not show positive effects of β-carotene when used as an extender supplement during cryopreservation of rainbow trout sperm. In fact, it was the only antioxidant used that decreased the post-thaw motility rate. According to Mortensen, et al. [89], dietary β-carotene can show pro-oxidative effects as well as antioxidation behavior, which could explain the previously mentioned results. On the other hand, a more natural source of carotenoids, *Spirulina*, rich in β-carotene, xanthophylls, zeaxanthin, echinenone, and cryptoxanthin, was successfully tested as a diet supplement and could enhance coloration, growth and reproduction performance on female yellow tail cichlid (*Pseudotropheus acei*) [103]. In view of the above, there is a wide spectrum of action for carotenoids to exploit regarding fish physiology, namely, their action at the sperm level and how they could prevent oxidative processes and cellular damage.

#### 3.2.6. Carnitines

Carnitine is a water-soluble ammonium compound, bio-synthetized from lysine and methionine, and a powerful antioxidant [104,105]. Carnitines, such as L-acetyl carnitine and L-carnitine, are naturally present in microorganisms, animals, and plants, and are responsible for fatty acid transport into the mitochondria for β-oxidation in order to generate ATP energy, reducing the availability of lipids for peroxidation [104]. In mammals, carnitines are known for their importance in sperm maturation and metabolism [37,106]. According to Elokil, et al. [107], L-carnitine has a powerful effect on sperm, being involved in the correct functioning of mitochondrial oxidation, preservation of membrane integrity (Figure 1), balance of sperm energy and inhibition of apoptosis. Moreover, an appropriate dietary level can promote growth performance and antioxidant status in fish [108]. In fact, there are studies in spermatozoa from human [46], stallion [109], buffalo [105], boar [110], duck [111], rooster [107,112], Japanese quail [113], rabbit [106] and rat [114], revealing the positive effects of L-carnitine, not only on sperm quality parameters, such as motility, viability, and cell concentration, but in some cases, it decreased sperm ROS production. Given the literature available, there are just a few studies in fish in which carnitine was used as a dietary supplement, testing its effect on sperm quality. From all species analyzed by Lahnsteiner and Mansour [27], carnitine was only detected on sperm and spermatozoa of bleak and, an in vitro experiment, showed that carnitine concentration of 0.5–2.0 mmol/L improved sperm motility, velocity, and membrane integrity, showing some species-specific effect. When it is naturally present, carnitine is also responsible for the improvement on the pyruvate usage, which is essential to prolong sperm motility and viability [115]. However, levels of lipid peroxidation were maintained on the in vitro assay, which led to the conclusion that more than acting as an antioxidant on bleak sperm, carnitine affected metabolism, possibly via mitochondrial functions [27]. Carnitine was also used in the extender media for rainbow trout sperm cryopreservation and it was able to improve post-thaw motility, although fertility was not affected by the supplement, the higher fertilization rate was obtained with samples cryopreserved with carnitine [29]. In other marine and freshwater species, such as the European seabass [116], largemouth bass (*Micropterus salmoides*) [117] and common carp [118], L-carnitine proved to have an impact on the growth performance and antioxidant status. Further studies are necessary to better comprehend the mechanisms of action of this compound, specially at fish spermatozoa level.

#### 3.2.7. Polyphenolic Antioxidants: Is There a Natural Solution?

Plants are the only organisms capable of synthesizing polyphenols [119], a very diverse group of antioxidants normally classified according to the number of phenol units present in their structure, which is determinant for the displayed antioxidant activity [13]. Polyphenols are classically subdivided in flavonoids and non-flavonoids, and both have several subgroups [120]. Independently of the group, most dietary polyphenols are multi-function antioxidants, not only due to the ability to neutralize free radicals, inhibiting lipid peroxidation, and reducing the cell level of ROS, but also to their ability of exerting many other bioactivities, like anti-inflammatory, anti-mutagenic, anti-carcinogenic, anti-allergic, metal chelation, among many others [27,33,120,121]. Depending on the compound and animal species, phenols can be more or less absorbed [30]. Within the category of polyphenols, non-flavonoids phenolic acids are the most applied to fish sperm cryobiology. A study performed by Osipova, et al. [121] with beluga sperm, showed that a higher percentage of fertilization was achieved when using the phenolic compound methylenediphosphonicacid (MDPA) as sperm cryoprotectant, revealing better results than the butylated hydroxytoluene (BHT) or Trolox. BHT is a lipophilic synthetic phenolic antioxidant widely used by the industry in the manufacture of plastics, oils, fragrances, lubricants, vitamin complexes, and as an additive to improve the shelf-life of some food products [122]. It has been supplemented in fish sperm cryopreservation extenders in order to protect the cellular function and structure [123] and showed to have a beneficial effect improving motility parameters, fertilization rate and eyed-egg rate in carp [122]. It was also used in sturgeon sperm, reducing lipid peroxidation, and enhancing motility [124]. Moreover, in short-term storage of coho salmon (*Oncorhynchus kisutch*) sperm, the incorporation of 2 mM of BHT in the extender medium not only improved motility and mitochondrial membrane potential, but also reduced the superoxide anion level in spermatozoa [123].

Different from the synthetics, many polyphenols that are naturally found in the human diet have been used for centuries to preserve foods because of their antioxidant proprieties [30], and have recently had their protection effect tested on cells exposed to oxidative processes, like cryopreservation. There are many examples of extracts from herbs and spices being applied to antioxidant experiments. Rosemary, rich in rosmarinic acid, has been successfully used in boar [63] and deer [125] sperm cryopreservation extender; green and white tea extracts, containing catechins, were applied to rat [126], human [127], and rabbit [128] reproduction systems; saffron attenuated oxidative damage in human sperm [129], and improved sperm quality and fertilization capacity in bovine thawed sperm [130]; curcumin, also known as turmeric, greatly improved human sperm quality and TAS [131], dog sperm protection against ROS [132], Angora goat (*Capra hircus ancryrensis*) morphology and motility [133] and rooster (*Gallus gallus domesticus*) sperm viability, motility and protection from ROS [134]. Some of the spice extracts mentioned above were also tested on fish reproductive cells: black pepper, rich in phenolic acid amides, and turmeric mended the impairment caused by cadmium on African catfish testicular tissues [135] while stressed Senegalese sole fed with paprika diet counteracted the induced oxidative stress effects on spermatozoa [136]. Others like ginger diet containing dehydrozingerone improved growth, feed conversion and protein efficiency in rainbow trout infected with *Aeromonas hydrophila* [137], and also immunological activity in Asian seabass (*Lates calcarifer*) infected with *Vibrio harveyi* [138], proving to be a great antioxidant and immunostimulant in aquaculture species [139]. However, further studies are necessary to test a possible effect of this compound in protecting spermatozoa from oxidative stress.

**Table 1:** Polyphenolic compounds used as antioxidants on target tissues of fish and mammals. CM—cryoprotectant medium, LPO—lipid peroxidation, SOD•—superoxide dismutase anion, FCR—feed conversion ratio, TAC—total antioxidant capacity, GR—glutathione reductase, CAT—catalase, MDA—malondialdehyde acid, ROS—reactive oxygen species, SOD—superoxide dismutase, GSH—glutathione.

	Phenolic Compound/Extract	Species	Target	Administration Mode	Main Effects	Ref
Fish	MDPA	Beluga sturgeon	Sperm	CM	↑ Fertilization rate	[121]
	BHT	Common carp	Sperm	CM	↑ Sperm motility, fertilization rate, eyed-egg rate	[122]
		Russian sturgeon	Sperm	CM	↑ Sperm motility, ↓ LPO,	[124]
		Coho salmon	Sperm	CM	↑ Sperm motility, mitochondrial membrane potential, ↓ SOD• in spermatozoa	[123]
	Curcumin + black pepper	African catfish	General health	Diet	↑ Growth, ↓ hepatotoxic, nephrotoxic, and reprotoxic effects of cadmium	[135]
	Curcumin	Sand goby	General health	Diet	↑ Digestive enzymes activity, growth	[140]
	Paprika	Senegalese sole	Sperm	Diet	↑ Sperm concentration, ↓ skin injuries	[136]
	Ginger	Rainbow trout	General health	Diet	↑ Growth, FCR and protein efficiency	[137]
		Asian seabass	General health	Diet	↑ Survival, growth, FCR, immunological activity	[138]
		Nile tilapia	General health	Diet	↑ Growth, blood plasma total proteins ↓ mortality, blood glucose, triglycerides, and cholesterol	[141]
	Blueberries	Artic char	Sperm	Diet	↑ CAT-like activity, ↓ LPO	[6]
	Sesame seed	African catfish	Reproductive system	Diet	↑ Sperm motility duration, hatchability, and egg survival rate	[142]
	*Gracilaria* sp.	European seabass	General health	Diet	↑ TAC, down-regulated heat shock proteins ↓ LPO, delayed mortality	[143]
	Propolis	Common carp	Sperm	CM	↑ Sperm integrity, motility, and hatchability	[144]
		Nile tilapia	Spleen	Diet	↑ Survival, number of lymphocytes, GR	[145]
		Rainbow trout	Brain	Diluted in water	↑ CAT, ↓ MDA	[146]
Mammals	Rosemary	Boar	Sperm	CM	↑ Sperm motility, fertilization capacity	[63]
		Deer	Sperm	CM	↑ Sperm motility, membrane integrity and live cells	[125]
	White tea	Rat	Sperm	Extender	↑ Sperm survival, ↓ LPO	[126]
	Green tea	Rabbit	General health	Oral	↑ Reproductive performance, lipid metabolism, preserve hematological parameters, kidney, and liver functions	[128]
		Human	Sperm	Extender	↑ Sperm motility, viability, phosphorylation of proteins, cell survival	[127]
	Saffron	Buffalo	Sperm	CM	↑ Sperm motility, viability, acrosome integrity, ↓ ROS, LPO	[130]
		Human	Sperm	Oral	↓ Oxidative damage on sperm DNA	[129]
	Curcumin	Human	Sperm	Oral	↑ TAC, ↓ MDA, C-reactive protein, tumor necrosis factor	[131]
		Dog	Sperm	CM	↑ Sperm DNA integrity, TAC, NOX-5 gene expression	[132]
		Angora goat	Sperm	CM	↑ Sperm motility, morphology, SOD activity	[133]
		Rooster	Sperm	Diet	↑ Sperm motility, viability, ↓ROS	[134]
	Murtilla	Boar	Sperm	CM	↑ Sperm motility, ↓ ROS, membrane damage	[147]
	Royal jelly	Buffalo	Sperm	Extender	↑ Sperm viability, membrane and acrosome integrity, fertilization capacity	[148,149]
		Goat	Sperm	Extender	↑ Sperm motility, membrane integrity ↓ acrosome damage	[150]
		Ram	Sperm	Extender	↑ Sperm motility, membrane integrity, cell viability	[151]
		Rat	Sperm	Oral	↑ Sperm motility, concentration, SOD, CAT and GSH activity ↓ Sperm abnormalities, MDA, apoptotic cells	[152]
	Propolis	Rat	Sperm	Oral	↑ Sperm motility, morphology, embryo development ↓ MDA	[153]
	Hazelnut	Rat	Sperm	Diet	↑ Plasma testosterone, plasma oxidant-antioxidant balance	[154]

All mentioned herbs and spices are thought to inhibit lipid peroxidation due to their phenols’ content [30]. Some fruits and seeds extracts have also been used on animal reproduction and antioxidant studies, including in fish. Extracts from berries (rich in quercetin and catechins) like murtilla [141] and blueberries proved to inhibit sperm lipid peroxidation in Arctic char [6], while sesame seed powder diet, containing lignans like sesamin, was able to improve sperm motility duration, hatchability, and egg survival rate in African catfish [142]. Even algae extracts of Gracilaria sp., containing phenolic compounds, proved to have an antioxidant effect on European seabass challenged with a bacterial infection, decreasing lipid peroxidation and suggesting higher total antioxidant capacity in fish fed with experimental diet [143]. Other polyphenolic substances that have been widely used in many different types of antioxidant assays are honeybee products such as honey, propolis, and royal jelly. Propolis is a resinous substance used by bees, rich in flavonoids, phenolic acids, steroids, and amino acids that is known for their antimicrobial, immunomodulatory, anti-inflammatory, detoxicant, antioxidant, antiapoptotic and other benefic proprieties in humans and animals, including protection of the male reproductive system [155,156]. There are many studies assessing the antioxidant effect of propolis and royal jelly on sperm protection. Experiments in buffalo [148,149], goat [150], ram [151] and rat [152,153], are examples of enhanced sperm motility, spermatozoa viability and antioxidant status. Although the effect of these extracts on fish spermatozoa was not so explored so far, results were surprisingly positive when propolis was added to common carp sperm extender and spermatozoa integrity, motility, and hatchability were enhanced [144]. Despite the investigation done so far (consult Table 1) more research is still needed to understand whether plant phenols act as anti or pro-oxidants in vivo [30].

#### 3.2.8. Low Molecular Weight Antioxidants

All plants and animals have GSH present in their cells, a low molecular weight tripeptide whose structure is composed by three amino acids: cysteine, glycine, and glutamic acid. It may be present in different redox forms, being the most representative of the reduced glutathione (normally represented by the general abbreviation GSH) and the oxidized glutathione (GSSG) [157,158]. The GSH redox form, concentration, and role are cell-type-dependent [13]. GSH is considered to be a multifunctional intracellular thiol antioxidant and a cell redox buffer [21]. It is involved in many metabolic functions, detoxification processes, transport of amino acids, absorption of micronutrients and, above all, it is a potent antioxidant [13]. Considering this last function, GSH is capable of reducing ROS, regenerating other antioxidant molecules, such as vitamin E and C, repairing proteins, nucleic acids, and lipids damaged by peroxidation [158]. Its role has also been pointed out in the participation of DNA-repair mechanism and it is involved in cell replication and growth [157]. The effect of GSH on fish sperm cryopreservation has been tested in several species. Lahnsteiner and Mansour [27] performed experiments using different forms of GSH and results differed considerably according to the species: on perch, GSH increased sperm motility and velocity, and GSSG increased sperm motility of the cyprinid bleak, and none of the redox forms of this molecule had effect on burbot and brown trout. The findings of Muthmainnah, et al. [159] with other cyprinid species, seurukan fish (*Osteochilus vittatus*), are in accordance with the previous results, in which different concentrations of GSH had positive effects on cryopreserved sperm motility, fertility, and hatching rate. Regarding salmonids results are inconsistent. Lahnsteiner, et al. [68] tested the effect of GSH and a mixture of reduced and oxidized forms on sperm cryopreservation of different species, brook trout, and rainbow trout. Results revealed that just the mixture had an impact on post-thaw sperm, but only on brook trout sperm velocity. In rainbow trout, contradictory results were found by Sarosiek, et al. [160], who reported a negative impact on cells by adding GSH to the extender medium of sperm from rainbow trout and Arctic char during short-term storage. Despite GSH being one of the antioxidants naturally present in salmonid testes [161], it seems that exogenous addition of this tripeptide is not enough to improve sperm protection during long or short-term storage procedures. However, different methodologies may lead to different results, as it happened with another study on rainbow trout, in which GSH revealed a positive effect on sperm motility when supplemented to the extender medium [29]. On the other hand, GSH was revealed to have a protective effect on sperm of different sturgeon species after submitted to cryopreservation procedures [25]. A study from Stejskal, et al. [65] determined the ratio of reduced and oxidized forms of this molecule in sperm from different teleost species, sturgeons, and cyprinids, and found that GSH and GSSG ratio is highly variable between species, being the content of GSH about three times higher than GSSG. In addition, this antioxidant ratio is normally used as a biomarker of cellular toxicity [162], and thus, measuring it to identify some imbalance can potentially predict oxidative stress in fish sperm. Once more, the species-specific effect is obvious regarding GSH, as it was demonstrated for other antioxidants.

Coenzyme Q10 (cQ10), also known as ubiquinone, is a low molecular weight cofactor synthetized in all tissues of the body that plays a crucial role in the mitochondrial electron transportation chain and participates in aerobic respiration [37]. Coenzyme Q can be found in vertebrates and mammals and is involved in several biochemical reactions. Additionally, it has proven to be very efficient in preventing proteins, lipids, and DNA oxidation [163]. Depending on its oxidation status, Coenzyme Q can take the name of ubiquinone or ubiquinol and displays different antioxidant proprieties and affinities for specific free radicals according to its structural form [13]. In humans, it is the only lipid-soluble antioxidant synthesized endogenously, it can reduce sperm oxidative stress, inhibit peroxide formation in seminal fluid and increase the overall seminal quality: motility, total sperm count, concentration and morphology [46]. Although, until now, no one has tested the protective effect of cQ10 in fish sperm during storage, there are few studies assessing its impacts in stallion [164], buffalo, cattle [165], and ram [166] sperm. Short- and long-term storage results seem to be similar with 1 µM cQ10 in stallion and ram sperm, improving spermatozoa quality traits, and reducing apoptotic-like changes and lipid peroxidation [164,166]. However, in two different species, cattle and buffalo, the best results were achieved with higher concentration of cQ10 (30 µM) [165]. The number of mitochondria in fish spermatozoa depends on the species, but are usually few with the capacity to restore ATP consumption after motility activation [12,167]. In fact, it would be interesting to find whether cQ10 could enhance the fish sperm quality parameters.

Uric acid (UA) is a low molecular weight organic compound produced from hypoxanthine and xanthine. Although UA is a hydrophilic antioxidant, it has limited solubility in water since high concentrations in fluids can lead to crystallization [13,30]. Also known as urate, it is a powerful scavenger of reactive species, including singlet oxygen species and lipid peroxides [13]. In humans, it is normally present intracellularly and in many body fluids, such as blood plasma, saliva, urine, milk, tear, and synovial fluids [30]. In fish, it is one of the main antioxidants present in blood plasma and, according to Ciereszko, et al. [168], it is also present in seminal plasma on a similar or higher level, depending on the species. Therefore, it is thought that UA has a considerable protective function at spermatozoa level against ROS. Lahnsteiner and Mansour [27] revealed that UA is the major antioxidant of seminal plasma present in different teleost species and has the ability to decrease sperm lipid peroxidation and improve sperm motility and membrane integrity when used at 0.5 mmol/L on cryopreservation mediums. The results obtained by these authors were consistent in all analyzed species from the families of Percidae, Salmonidae, Cyprinidae, and Lotidae and are in accordance with previous results from Ciereszko, et al. [168], wherein he affirms that UA is present at high concentrations in seminal plasma of rainbow trout, yellow perch, muskellunge (*Esox masquinongy*), Northern pike (*Esox lucius*), common carp, common bream (*Abramis brama*), and tench (*Tinca tinca*). Furthermore, the same analysis was performed on brown trout sperm, and results were coherent regarding the presence of high levels of UA in seminal plasma and its positive effect on spermatozoa quality in vivo and post-thaw, protecting cells from lipid peroxidation [17]. Nevertheless, other studies using sperm samples from rainbow trout revealed a positive effect of UA in spermatozoa protection against cryopreservation damage, improving sperm motility rate and movement duration, but no effect was observed regarding fertilization and hatching rates [29]. As other mentioned antioxidants, UA is not an exception, and its levels on seminal plasma are species-specific [168]. Moreover, Ciereszko, et al. [168] found that concentrations of UA in rainbow trout urine were significantly lower than in seminal plasma, suggesting that small contamination with urine during stripping cannot affect the UA seminal plasma concentration, which has a protective function against oxidative damage. Although it is widely seen as a powerful antioxidant and ROS scavenger, there are also some studies indicating pro-oxidant proprieties [13], but none of those results have been obtained in fish sperm. Further studies are still necessary regarding the effect of UA to completely understand its mechanism and function at the sperm level in different species. The same applies to lipoic acid, which, as far as we know, there is only one publication about its effects on fish sperm. Inanan and Kanyilmaz [169] tested different concentrations of α-lipoic acid (ALA) on common carp sperm extender during short- and long-term storage. The optimal concentration of ALA that confers proper protection to common carp spermatozoa in terms of lipid peroxidation and viability is 0.5 mM for short-term storage and 1 mM for cryopreservation techniques, both improving motility parameters. ALA is a natural thiol antioxidant, both hydro and lipophilic, as a result, it can be found both in cellular membranes and in cytosol [158]. It is readily absorbed by the organism and its reduced form, dihydrolipoic acid (DHLA) is an antioxidant as powerful as ALA and can act synergically with other antioxidants, such GSH, ascorbate, and tocopherol [21]. Although levels of either ALA or DHLA are low in animal tissues and fluids, they are able to scavenge free radicals, metal ion chelation, recycle antioxidants, and repair protein damage caused by oxidative stress [30]. For this reason, it should be interesting to validate the existing results on other species and investigate its interactions with other antioxidants at fish spermatozoa level.

Melatonin (MEL) is a common molecule in nature that evolved through billions of years in all taxa and, against evolution odds, maintained its chemical structure until now [170]. Primordially, its antioxidant capacity was essential to cyanobacteria to scavenge the free radicals produced by photosynthesis [171], but through diversification of species, MEL not only retained its capacity to control oxidative stress in all animals and plants, but also assumed many other functions: it is a sleep regulator, controls the biological circadian rhythms, enhances immunity and, more recently, it appears as a multifunctional oncostatic agent [172]. It is considered a potent antioxidant because it acts on both aliphatic and aqueous cell environments with the capacity to detoxify ROS and RNS and regulates other antioxidant enzymes [13]. Moreover, MEL can up-regulate anti-apoptotic genes (*bcl2l1* (*bcl-xL* family) and *bcl-2*) and down-regulate pro-apoptotic genes (*bax*) (Figure 1) [15], which is crucial to prevent cellular apoptosis. This indolamine was first thought to be produced only by the pineal organ, but nowadays, it is known that MEL is also produced by many organs and tissues, being mitochondria one of the main producing organelles, reaching concentrations even higher than in bloodstream [173,174].

### 3.3. Potential Role of Melatonin

#### 3.3.1. Melatonin Production Sites on Fish

Although the MEL pathway varies between species, the precursor tryptophan seems to be transversal to all [172]. There are two enzymatic processes involved in MEL production: the first one is serotonin formation from tryptophan, and the second one is the conversion of serotonin into MEL, through the action of two different enzymes, arylalkylamine N-acetyltransferase (AANAT) and hydroxyindole-O-methyltransferase (HIOMT) [10]. Noteworthy, teleost fish have two AANAT genes, AANAT1 is expressed in the retina and brain, and AANAT2 is expressed in pineal organ [10]. MEL production by the pineal organ is controlled by the dark/light cycle, being produced during the dark phase of photoperiod, and inhibited by light, which induces a decrease in AANAT2 activity. Contrarily, during night period, photoreceptor depolarization allows Ca^2+^ entry through its channels and cAMP accumulation, both contributing for the increase of AANAT2 activity and phosphorylation of AANAT2 protein [175], and leading to MEL production. Also, in retina, MEL is produced during the night, but not all fish species seem to follow the same behaviour in this regard [176,177]. Zebrafish [178] and goldfish [179] have ocular melatonin production during the night, but other species like rainbow and brook trout [180,181] clearly showed an opposite pattern. MEL from retina is the exception to the rule, once its peaks depend on species and time of the year [180,182]. There are also reports of gastrointestinal MEL production in many species, including goldfish [183], great lake sturgeon (*Acipenser fulvescens*), rainbow trout, and common carp species [184], wherein levels were even higher than the average in the blood plasma concentration. The measurement of MEL subcellular distribution led to the discovery of MEL production by mitochondria, wherein it is likely to exert a direct free radical scavenger activity due to the accumulation of toxic metabolites from oxygen production [172]. Thus, under oxidative stress, MEL improves mitochondrial respiration, increases ATP synthesis, prevents mitochondrial membrane depolarization and the ultimate opening of mitochondrial permeability transition pore (MPTP), which triggers apoptosis [185,186]. In fish, MEL is known to be produced in the pineal organ, retina, gastrointestinal tissues [10], but since it is produced in mitochondria, it may be present in many other tissues, uncovering an organism melatonin concentration much higher than thought until now [172]. The endosymbiotic theory of the origin of mitochondria supports the idea of the production of MEL by mitochondria itself, rather than only be taken up from the circulation by cells [170]. Moreover, ophthalmectomy and pinealectomy induced species-specific alterations on circadian activity of fish, which is an evidence of a robust and organized MEL production network located in different extra-pineal and extra-retinal areas [10].

#### 3.3.2. Melatonin-Mediated and Non-Mediated Mechanisms and Targets

MEL is a hormone that functions via receptor-dependent and independent ways [172]. As part of the low molecular weight group, MEL has a small molecular size and a simple steroid chemical structure, which confers the ability to permeate through membranes and act directly on target components of the cell [13]. Moreover, autocrine signaling has been described as MEL mechanism of action in some organs and tissues, which are the source and target of MEL, like in retina [187] and mitochondria, wherein paracrine regulation is also described [188]. But this is not the only mechanism used by this indole. Mayo, et al. [189] reviewed the different MEL uptake strategies by cells and despite passive diffusion, protein-facilitated transport is also highlighted, specifically through the glucose transporters (GLUT/SLC2A). However, it remains to be answered whether these two systems are compatible and if a specific strategy is activated upon a specific circumstance or cell type. The effects of MEL are mostly described as mediated through receptors. In fish, three high affinity receptor subtypes were identified: MT1 (Mel1a), MT2 (Mel1b), and MT3 (Mel1c), all belonging to the seven transmembrane G-protein-coupled receptor (GPCR) family [190]. The first two are widely distributed in brain, retina, and peripheral tissues, while Mel1c is mainly present in skin and traces were found in retina [187]. Normally, each receptor is coupled to intracellular pathways and fish MT2 is negatively coupled with cAMP pathway [191]. In addition, expression of these receptors has been detected in other fish tissues, like kidney, liver, gills, gonads and blood cells [10,188,192], which opens up the possibility for new discoveries regarding MEL targets and functions on fish species.

#### 3.3.3. Different Methods for Spermatozoa Protection

Photoperiod and MEL production by fish pineal organs have an important role in testicular maturation [11], yet results are not always consistent because they vary within species, gender, light regime, and reproductive phase [182]. Generally, the nocturnal MEL peak regulates the GnRH secretion by the hypothalamus via kisspeptin [193], which controls lutein hormone (LH) release, the hormone responsible for the male sperm production [192], like in masu salmon (*Oncorhynchus masou*) [194] and Indian carp (*Catla catla*) [195]. Although it is not clear yet if it comes from pineal organ or if it is locally produced, MEL has been reported to have an impact on spermatozoa protection and quality traits improvement. However, there is only limited and recent literature regarding the effects of MEL on fish reproductive performance and sperm quality protection against oxidative damage, both in vivo and in vitro. A study on killifish (*Fundulus heteroclitus*) proved that fish exposed (via water) for 8 days to 1 µM MEL improved its sperm motility and velocity parameters, and these results were in accordance with the fertilization ratios and embryos survival experiments [190]. An in vitro incubation of long-spine scraper trout (*Capoeta trutta*) sperm to noxious titanium dioxide nanoparticles (TiO_2_-NPs) found that 0.1 mM and 1 mM MEL can reverse the toxic effects of the nanoparticles on sperm, improving spermatozoa velocity parameters (VSL, VCL and VAP) and preventing lipid peroxidation [196]. Among other mitochondria-targeted antioxidants, MEL can also be used on assisted reproductive techniques (ART), like cryopreservation, to prevent damage associated with reactive species, although its specific mechanism of action is still unclear [15,170]. Gao, et al. [197] evaluated the effects of different MEL concentrations during sperm storage of paddlefish (*Polyodon spathula*) at 4 °C and 0.5 µM MEL showed the best results: in non-activated sperm, it improved plasma membrane integrity, maintained ROS levels, and inhibited the decrease of mitochondria membrane potential. In what activated sperm is concerned, it improved sperm motility parameters (TM, PM, BCF), motility duration and maintained high content of ATP in spermatozoa before and after activation. A different study using MEL implants showed that this hormone has a long-term negative effect on gonadogenesis in European seabass affecting testicular maturation, percentage of running males during spermatogenesis and plasma androgen levels during the reproductive season, possibly, due to a downregulation of kisspeptin-GnRH activity on fish brain, induced by MEL [198]. However, the opposite effect was observed in zebrafish females, wherein MEL induced gametogenesis and stimulated follicle maturation [199], and on Indian carp [200], in addition, MEL acted as an antioxidant and reduced oxidative stress during follicle maturation. These results are proof of MEL variability response among different fish species, as it happens with all reviewed antioxidants. As an example, in other freshwater species, curimba (*Prochilodus lineatus*), MEL was used to supplement the sperm cryopreservation medium and no significant differences were obtained among different concentrations (1, 2, and 3 mM MEL) neither regarding motility parameters, spermatozoa morphological anomalies nor fertilization capacity [201]. Noteworthy, MEL can exert its effect in a dose-dependent manner [202] or depending on the administration method. Supplementing feeds with MEL was also tested by Aripin, et al. [203], which enhanced testes and sperm maturation on the first puberty event of walking catfish (*Clarias macrocephalus*). Furthermore, on the same species and through feed supplementation as well, MEL associated with zinc improved sperm motility, gonadosomatic index (GSI), and decreased sperm abnormality [52]. Different approaches to expose sperm to MEL have been referred: via water dilution, added to the cryoprotectant medium, through implants or feed supplementation, but MEL injections [204] is also an effective method that can be applied. It is also important to refer that an excellent antioxidant in vitro may not work in vivo: it may not reach the target, it may be metabolized into inactive products, or rapidly excreted by the organism [30], which can also explain some differences on the results presented here. Nonetheless, it is undeniable that effects of MEL on fish spermatozoa protection are promising, especially taking into consideration the gene regulation capacity of this molecule at the apoptosis level, which deserves scientific attention and, especially, could be more explored during spermatozoa long-term storage.

## 4. Conclusions

This analysis of literature regarding the effect of antioxidants on fish spermatozoa protection systematizes the vast information already available in the scientific community and provides questions for further investigation. Which kind of different antioxidants can be tested? Could we replace them with natural compounds? Which are their mechanisms of action? Other aspects mentioned above like antioxidants bioavailability, synergic effects, and inter and intra-species variability also need to be taken into consideration and explored when searching for antioxidants’ effects and efficiency on fish sperm protection against oxidative damage. Independently of the antioxidants studied, it is also important to avoid the “antioxidant paradox” [37] through the abuse of their administration. In the face of the above findings, future research should be conducted on the improvement of mechanisms for the enhancement of endogenously produced antioxidants, like melatonin, to naturally protect spermatozoa from oxidative events. To achieve this goal, more fundamental research is needed on melatonin insights at an intracellular level. This review aims to encourage the research on this emergent field to understand how we could control the inevitable spermatozoa oxidative processes and their consequences.
